# TBOPP, a DOCK1 Inhibitor, Potentiates Cisplatin Efficacy in Breast Cancer by Regulating Twist-mediated EMT

**DOI:** 10.2174/0115680096281231240202073558

**Published:** 2024-02-26

**Authors:** Xin Chen, Zhenbang Zhou, Pengting Tang, Feiya Du, Shuqian Wang, Jia Yao, Shufen Zhang, Jiajing Huang, Xuemei Lu, Wei Chen, Xiaofang Yu, Yu Liu, Hao Liu

**Affiliations:** 1 Department of Surgery, Women’s Hospital, School of Medicine, Zhejiang University, Hangzhou, Zhejiang, 310006, China;; 2 Cancer Institute (Key Laboratory of Cancer Prevention and Intervention, China National Ministry of Education), The Second Affiliated Hospital, Zhejiang University School of Medicine, Hangzhou, Zhejiang, 310009, China;; 3 Department of Surgery, Ninghai Maternity and Child Health Hospital, Ninghai, Zhejiang, 315600, P.R. China;; 4 Department of Orthopaedics, the First Affiliated Hospital, School of Medicine, Zhejiang University, Hangzhou, 310003, China;; 5 Department of General Surgery, the First Affiliated Hospital, School of Medicine, Zhejiang University, Hangzhou, 310003, China;; 6 Cancer Institute of Integrated traditional Chinese and Western Medicine, Key Laboratory of Cancer Prevention and Therapy Combining Traditional Chinese and Western Medicine of Zhejiang Province, Zhejiang Academy of Traditional Chinese Medicine, Tongde Hospital of Zhejiang Province, Hangzhou, 310012, Zhejiang Province, China;; 7 Cancer Center, Zhejiang University, Hangzhou, Zhejiang, 310058, China

**Keywords:** Breast cancer, cisplatin, DOCK1,TWIST 1, epithelial-mesenchymal transition, TBOPP

## Abstract

**Background:**

DOCK1 has been reported to be involved in tumor progression and resistance.1-(2-(30-(trifluoromethyl)-[1,10-biphenyl]-4-yl)-2-oxoethyl)-5-pyrrolidinylsulfonyl2(1H)- pyridone (TBOPP) is a selective DOCK1 inhibitor; however, the role and molecular mechanisms of DOCK1 and its inhibition in breast cancer (BC) resistance remain poorly understood. Objective: This study aims toinvestigate the underlying mechanisms of DOCK1 in BC resistance.

**Methods:**

DOCK1 or Twist siRNA and Twist plasmid were used to explore the function of DOCK1 *in vitro* experiments. A mouse xenograft model was used for *in vivo* experiments.

**Results:**

In the present study, we demonstrated that DOCK1 siRNA promoted cisplatin sensitivity in BC cells. Moreover, TBOPP also enhances the therapeutic effect of cisplatin both *in vitro* and *in vivo*. Mechanistically, DOCK1 siRNA inhibited EMT. Twist 1 is one of the EMT-inducing transcription factors and is known to induce EMT. To further reveal the effect of DOCK in BC cells, we co-transfected with DOCK1 and Twist1 siRNA to BC cells and found that co-transfection with DOCK1 and Twist siRNA could not further enhance the cisplatin sensitivity of BC cells. Moreover, DOCK1 siRNA failed to reverse the effect of Twist 1 up-regulation.

**Conclusion:**

Taken together, these results demonstrate that DOCK1 may function as a potential therapeutic target in BC and that combining cisplatin with TBOPP may provide a promising therapeutic strategy for cisplatin-resistant BC patients.

## 1 INTRODUCTION

Breast Cancer (BC) ranks second in female-related cancer deaths and substantially threatens the physical and mental health of women worldwide [1]. Currently, surgery and chemotherapy are the primary means of treating BC. Chemotherapeutic drugs (*e.g.,* cisplatin) are generally used to treat a variety of malignancies, including lung, ovary, and BC [2-4]. However, not all patients benefit from treatment, and some patients with BC develop cisplatin resistance [5]. Thus, there is a critical need to further study the molecular mechanism of drug resistance to cisplatin.

The Dedicator Of Cytokinesis (DOCK) family is a type of guanine nucleotide exchange factor and has been proven to regulate cell motility, survival, proliferation, and tumorigenesis [6-8]. DOCK1 (also known as DOCK180) was the first identified member of the DOCK family and can specifically bind and activate RAC11 [6]. A series of studies have demonstrated that DOCK1 is correlated with malignant phenotypes (*e.g.,* invasive, migration and metastasis). For example, DOCK1 inhibition can prevent malignant behaviors by harboring Rac1^P29S^ mutations in human cancer cells [9]. The downregulation of Dock1 and Elmo1 can suppress the migration and invasion of MDA-MB-231 cells and decrease Rac1 activity [10]; however, few studies have investigated the relationship between DOCK1 and drug resistance.

The Epithelial-mesenchymal Transition (EMT) is a process through which epithelial cells lose their properties and acquire a mesenchymal phenotype *via* the upregulation of Vimentin expression and down-regulation of the E-cadherin expression [11, 12]. EMT is a crucial mechanism for cancer metastasis, which promotes the migratory and invasive properties of cancer cells [13]. EMT is also considered to be a key step in the process of chemotherapy resistance. In cancer cells, EMT activation promotes migration, invasion and drug resistance, which can be reversed by EMT inhibition [14]. For example, in bladder cancer, DOCK1 downregulation enhances cisplatin sensitivity by suppressing EMT [15]. Numerous studies have shown that targeting Twist1 could significantly inhibit tumor growth, limit tumor metastasis, reverse drug resistance, and thus improve survival rates for cancer patients [16]. Twist 1, is the EMT-inducing transcription factor that plays a vital role in some physiological processes involving metastases [17]. Over-expression of Twist1 was related to resistance to both conventional chemotherapy and target agents in many cancer cell types [16, 18, 19]. Inhibition of Twist completely blocked EMT, partially reversed Multidrug Resistance (MDR) *in vitro*, and improved the efficacy of Adriamycin for BC *in vivo* [20]. It has been reported that Rab31 promotes metastasis and cisplatin resistance in stomach adenocarcinoma through Twist1-mediated EMT [21]. Therefore, there is a need to study the role of DOCK1 and EMT in BC drug resistance.

1-(2-(30-(trifluoromethyl)-[1,10-biphenyl]-4-yl)-2-oxoet-hyl)-5-pyrrolidinylsulfonyl2(1H)-pyridone (TBOPP) is a selective DOCK1 inhibitor first identified by Tajiri *et al.* [22]. The TBOPP-mediated cisplatin sensitizing effect was mediated by DOCK1 inhibition [23]. A recent study demonstrated that combined metformin and DOCK1 inhibition may provide a potential individualized therapeutic strategy for metformin-resistant liver cancer patients [24]. These results suggest that TBOPP can enhance the efficacy of chemotherapy; however, the underlying mechanism remains unclear. Therefore, our study aimed to explore the role and mechanism of DOCK1 and TBOPP in the process of cisplatin resistance in BC.

## 2 MATERIALS AND METHODS

### 2.1 Cell Culture and Transfection

Human BC cell lines were acquired from ATCC (Manassas, VA, USA). MDA-MB-468 and MDA-MB-231 cells were maintained in DMEM containing 10% FBS (Gibco) and 1% penicillin/streptomycin. MCF-7 cells were cultured in RPMI-1640 medium supplemented with 10% FBS. MCF-10A cells were cultured in DMEM/F12 supplemented with 5% horse serum, 20 ng/mL EGF, 0.5 mg/mL hydrocortisone, 100 ng/mL cholera toxin, 10 μg/mL insulin, and 1 × antibiotic-antimycotic (Gibco, Grand Island, NY). All cells were cultured in a humidified 5% CO_2_ atmosphere at 37°C. For the cell transfection assay, DOCK1 siRNA, Twist siRNA, or negative siRNA were synthesized by Santa Cruz Biotechnology (CA, USA). Twist plasmid was obtained from OriGene Company (MR227370). Transient transfection was performed using Lipofectamine 2000 Transfection Reagent (ThermoFisher Scientific, Waltham, MA, USA) according to the manufacturer's protocol.

### 2.2 Western Blot

The BC cells were lysed in a RIPA assay lysis buffer (Beyotime Biotechnology, Shanghai, China). The protein concentrations were quantified using a bicinchoninic acid kit (Beyotime), and 40 μg proteins were separated by 10% SDS-PAGE. The proteins were subsequently transferred to PVDF membranes, followed by incubation with 5% non-fat dry milk for 1 h at room temperature. The membranes were incubated with diluted primary antibodies against DOCK1 (#4846, Cell Signaling Technology, Danvers, MA, USA), vimentin (#5741, CST), E-cadherin (#3195, CST), Twist (#46702S, CST), and GAPDH (#5174, CST) overnight at 4°C. Horseradish peroxidase (HRP)-conjugated goat anti-rabbit IgG (#7074, 1:2000, CST) was used as a secondary antibody for 2 h. The protein bands were visualized with a Pierce™ ECL kit (Thermo Fisher Scientific).

### 2.3 Cell Viability Assay

Cells (5×10^3^ cells/well) were cultured overnight in 96-well plates. Untreated or TBOPP-treated cells were treated with different concentrations of cisplatin for 48 h. In the siRNA transfection studies, the cells were transfected with siRNA or plasmid prior to cisplatin treatment. A cell counting kit-8 (CCK-8) assay (Dojindo, Kumamoto, Japan) was used to detect cell viability. Briefly, CCK-8 solution (100 µL/well) was added to cells for 1-3 h at 37°C. The absorbance value was determined at a wavelength of 450 nm using an MRX II microplate reader (Dynex, Chantilly, VA, USA).

### 2.4 EdU Assay

In the cell proliferation assay, the cells were stained using a Click-iT EdU Imaging Kit (Invitrogen, Carlsbad, CA, USA; C10337) in accordance with the manufacturer’s instructions (ThermoFisher Scientific). Briefly, the transfected cells were incubated with cisplatin for 48 h. The cells were fixed in 1 mL of 3.7% formaldehyde in PBS for 15 min, followed by an incubation with 0.5% Triton X-100 for 20 min. Next, 0.1 mL of EdU reagent was added to the cells for 30 min, and the nuclei were stained with DAPI for 20 min at room temperature in the dark. The images were obtained under an inverted fluorescence microscope (Olympus; IX73).

### 2.5 Quantitative Real-time Reverse-transcription Polymerase Chain Reaction (qRT-PCR)

Total RNA was extracted with TRIzol reagent (Invitrogen, Carlsbad, CA, USA; 108-95-2), and the RNA (1 µg) was reverse transcribed using a cDNA synthesis kit (Takara, Kyoto, Japan; RR047A) according to the manufacturer’s instructions. A qRT-PCR assay was conducted with a Takara TB GREEB Premix Extaq kit (Takara; RR420A) using Applied Biosystems (Applied Biosystems, CA, USA). GAPDH was used to normalize the level of mRNA expression. The related sequence primers were as follows:

Twist 1

Forward: 5’-GCCGGAGACCTAGATGTCATT-3’

Reverse: 5’- TTTTAAAAGTGCGCCCCACG-3’

Notch1

Forward: 5’- AAGCTGCATCCAGAGGCAAAC-3’

Reverse: 5’- TGGCATACACACTCCGAGAACAC-3’

Stat3

Forward: 5’- CAACAGATTGCCTGCATTGGA-3’

Reverse: 5’- ATTTGTTGACGGGTCTGAAGTTGA -3’

ZEB1

Forward: 5’- ACTGTGGTAGAAACAAATTCAGATT-3’

Reverse: 5’- CCTTTCCTGTGTCATCCTCCC-3’

FOXC2

Forward: 5’- CTGTGCTGTGCTCGACCTGA-3’

Reverse: 5’- CATTTGTACAGCACGGTTGGAGA-3’

FOXF1

Forward: 5’- TTCTATTCCAGTTCCCGGCTA-3’

Reverse: 5’- AACCAGGTTCAGAGACTCACGAC-3’

FOXQ1

Forward: 5’- TTTAACGCCCAGGCTTCGTC-3’

Reverse: 5’- ACCTGGAAATGCCACATACGTACA-3’

Snail 1

Forward: 5’- GACCACTATGCCGCGCTCTT-3’

Reverse: 5’- TCGCTGTAGTTAGGCTTCCGATT-3’

Snail 2

Forward: 5’- TTTCCAGACCCTGGTTGCTTC-3’

Reverse: 5’- CTCAGATTTGACCTGTCTGCAAATG-3’

HMCA1

Forward: 5’- AAACAAGGGTGCTGCCAAGAC-3’

Reverse: 5’- TCCGAGGACTCCTGCGAGAT-3’

GAPDH

Forward: 5’- ATCATCAGCAATGCCTCC-3’

Reverse: 5’- TCCTTCCACGATACCAAAG-3’

### 2.6 Flow Cytometry

After treatment, the cells were collected, washed, and re-suspended in PBS to a concentration of 5 × 10^5^ cells/mL. An Annexin V-FITC/PI kit (BD Biosciences, San Jose, CA, USA) was used to measure cellular apoptosis on a BD FACSCanto™ II system. All of the results were analyzed with FlowJo software.

### 2.7 Tumour Xenografts

Animal experiments were performed according to the Guide for the Care and Use of the Animal Ethics Committee of the First Affiliated Hospital of Zhejiang University. Four-week-old male BALB/c nude mice were purchased from the Shanghai Experiment Animal Center (Shanghai, China). MDA-MB-231 cells (1 × 10^6^) were subcutaneously injected into the right armpit. The mice were divided into four groups based on treatment (n = 8 mice per group): control, cisplatin, TBOPP, and TBOPP plus cisplatin. Tumor length (L) and width (W) were measured every two days, and the tumor volumes were calculated according to the formula (L × W^2^)/2. The mice were scarified after two weeks of treatment, and the tumors were collected for subsequent experiments.

### 2.8 Statistical Analysis

The results were displayed as the mean ± SD. GraphPad 6.0 statistical software (San Diego, CA, USA) was used to conduct the statistical analysis. Statistical significance was examined using a Student’s t-test or one-way analysis of variance (ANOVA). All the experiments were repeated three times *in vitro*. Differences were deemed significant at *P <* 0.05.

## 3 RESULTS

### 3.1 A DOCK1 Knockdown Enhances BC Cell Sensitivity to Cisplatin

**Fig. (1):**
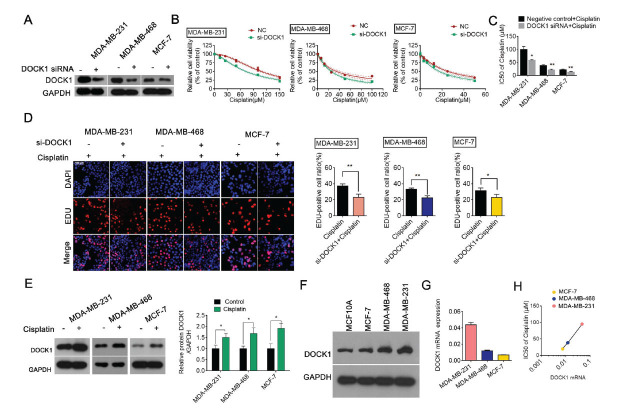
DOCK1 knockdown enhances breast cancer cell sensitivity to cisplatin. (**A**) Validation of DOCK1 knockdown efficiency following the transfection of breast cancer cells with DOCK1 siRNA or negative control, respectively. (**B** and **C**) Cisplatin sensitivity of breast cancer cells transfected with DOCK1 siRNA or negative control, as measured by a CCK-8 assay. The IC_50_ value of the cells was calculated following DOCK1 inhibition. *P < 0.05; **P < 0.01. (**D**) Cellular proliferation was measured using EdU staining and the EdU-positive cell ratio was calculated. (Scale bar: 100 μm). *P < 0.05; **P < 0.01. (**E**) A Western blot analysis was performed to evaluate the level of DOCK1 expression in cisplatin-treated breast cancer cells. *P < 0.05. (**F** and **G**) The level of DOCK1 protein and mRNA expression was detected in breast cancer cells *via* Western blot and qRT-PCR. (**H**) The IC_50_ of cisplatin was positively correlated with the level of DOCK1 mRNA in three breast cells.

To explore the functional role of DOCK1 in cisplatin resistance, we established DOCK1-deleted cells by transfecting BC cells with DOCK1 siRNA. Negative siRNA served as a negative control (NC). CCK-8 and EDU assays were performed, and the knockdown efficacy of DOCK1 was evaluated by Western blot (Fig. **1A**). The CCK-8 assay showed that the cell viability in the two groups gradually decreased in a dose-dependent manner. The cell viability in the DOCK1 siRNA group was lower than in those transfected with negative siRNA (Fig. **1B**). IC_50_ values (μM) further confirmed this result (Fig. **1C**). Compared with the NC group, DOCK1 down-regulation suppressed cellular growth under cisplatin treatment, as observed by a decreased EDU-positive cell ratio (Fig. **1D**). These results suggest that a DOCK1 knockdown enhances the sensitivity of BC cells to cisplatin; however, whether cisplatin affects the level of DOCK1 remains unknown. We performed a Western blot or RT-qPCR to examine the level of DOCK1 expression following cisplatin treatment. The results indicated that cisplatin treatment could increase the expression of DOCK1 protein and mRNA level, indicating that cisplatin resistance might be associated with DOCK1 (Fig. **1E** and Fig. **S1A**). Moreover, we detected the basic level of DOCK1 in three cell lines. The level of DOCK1 mRNA and protein expression differed in each of the three BC cell lines. DOCK1 expression was the highest in MDA-MB-231 cells, followed by MDA-MB-468 and MCF-7 cells (Figs. **1F**-**G**). Interestingly, BC cells with high DOCK1 expression exhibited greater drug resistance (Fig. **1H**). Taken together, the above results indicate that the DOCK1 knockdown sensitized BC cells to cisplatin and cisplatin resistance was correlated with DOCK1 expression.

### 3.2 TBOPP Enhances the Sensitivity to Cisplatin *In vitro* and *In vivo*

**Fig. (2):**
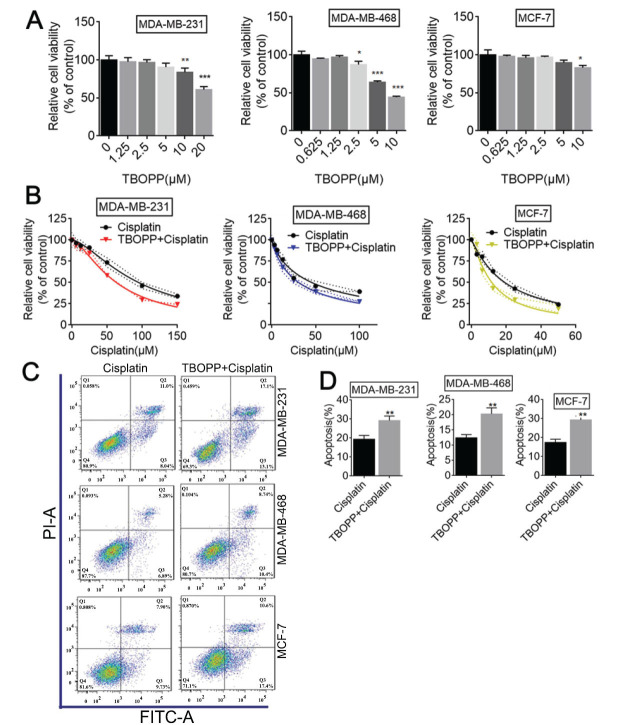
TBOPP enhances cisplatin sensitivity in BC cells. (**A**) BC cells were treated with various concentrations of TBOPP(μM) (0, 1.25, 2.5, 5, 10 in MDA-MB-468 and MCF-7 cells or 0, 1.25, 2.5, 5, 10, 20 in MDA-MB-231 cells) for 48 h. Cellular viability was evaluated by a CCK-8 assay. (**B**) The cell viability was measured by a CCK-8 assay after treated with Cisplatin or combined with TBOPP in BC cells. (**C** and **D**) The cell apoptosis ratio was measured and calculated after treated with Cisplatin, or combined with or without TBOPP using flow cytometry. **P < 0.01.

**Fig. (3):**
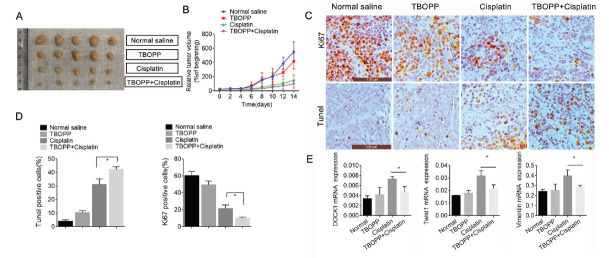
TBOPP can effectively enhance the effect of cisplatin on BC *in vivo*. (**A**) Representative images of xenograft tumors in BALB/c nude mice after different groups of treatment (Normal group, TBOPP group, Cisplatin group or TBOPP+ Cisplatin group). (**B**) The Growth curves of xenograft tumors in each group was statistically analyzed in 0 days to 14 days. (**C** and **D**) The Ki-67 expression and cell apoptosis in each group was detected by immunohistochemistry and a TUNEL assay, respectively. *P < 0.05. (**E**) The expression of DOCK1, Twist and Vimentin was measured by qRT-PCR in Normal group, TBOPP group, Cisplatin group or TBOPP+ Cisplatin group. *P < 0.05; ** *P <* 0.01.

Next, we investigated whether a DOCK1 inhibitor had similar effects to DOCK1 siRNA in cisplatin resistance. First, the level of TBOPP cytotoxicity in BC cells was measured by CCK-8. The results showed that TBOPP had virtually no effect on cell viability in MDA-MB-231 and MCF-7 cells between 0 and 5 μM, and MDA-MB-468 between 0 and 1.25 μM, indicating that TBOPP had little cytotoxicity against BC cells at low concentrations (Fig. **2A**). Conversely, cell viability was significantly inhibited when the concentration of TBOPP exceeded 10 μM in MDA-MB-231 and MCF-7 cells or exceeded 2.5 μM in MDA-MB-468 cells (Fig. **2A**). We further determined that,1.25 μM TBOPP was selected for further experiments. When cisplatin was combined with TBOPP treatment, TBOPP could enhance the therapeutic effect of cisplatin in BC cells, as evidenced by decreased cell viability and increased cell apoptosis (Figs. **2B**-**D**). We also determined the cell viability in normal Breast cells MACF-10A after treatment with different concentrations of TBOPP, and further verified that there was no significant difference in cell viability between the Cisplatin group and Cisplatin combined with TBOPP group in MCF-10A cells (Fig. **S1B** and **C**). Furthermore, a xenograft mouse model demonstrated that the tumor size was further reduced in the TBOPP and cisplatin combination group compared with the cisplatin group (Fig. **3A**). Fig. (**3B**) reflects that the TBOPP and cisplatin combination group had the slowest growth in tumor volume. Moreover, when combined with cisplatin, TBOPP could further suppress cellular proliferation and enhance apoptosis, as revealed by an obvious reduction in Ki67-positive cells and a significant increase in TUNEL-positive cells (Fig. **3C** and **D**). Compared with the cisplatin group, TBOPP administration could inhibit DOCK1 and EMT (Fig. **3E**). All of the above results demonstrate that TBOPP enhances the sensitivity to cisplatin *in vitro* and *in vivo*.

### 3.3 DOCK1 Knockdown Inhibits EMT

**Fig. (4):**
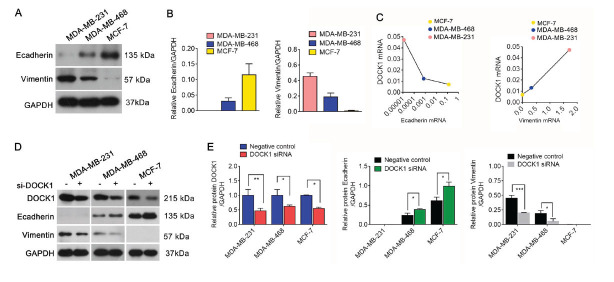
The effect of DOCK1 on EMT. (**A** and **B**) The level of E-cadherin and Vimentin protein expression was detected in BC cells *via* Western blot. (**C**) DOCK1 was negatively correlated with the level of E-cadherin mRNA and was positively correlated with the level of Vimentin mRNA in BC cells. (**D** and **E**) Western blot analyses were performed to evaluate the level of DOCK1, Vimentin, and E-cadherin expression in BC cells transfected with DOCK1 siRNA or NC, respectively. *P < 0.05; **P < 0.01; ***P < 0.001.

**Fig. (5):**
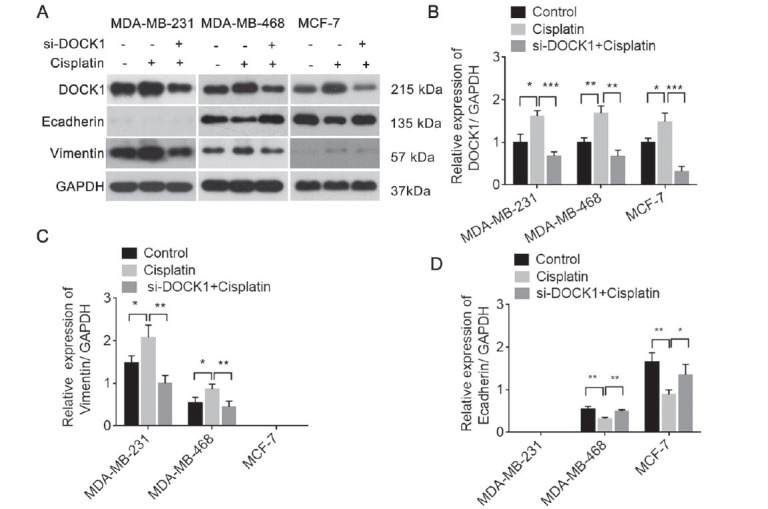
The effect of DOCK1 knockdown on EMT in the cisplatin-treated breast cancer cells. (**A-D**) A Western blot was used to detect the level of DOCK1, E-cadherin, and vimentin expression. *P < 0.05; **P < 0.01; ***P < 0.001.

Given that EMT plays an important role in drug chemotherapy, we assessed the level of E-cadherin and vimentin protein expression in BC cell lines under baseline conditions. We found that MCF-7 cells exhibited the highest levels of E-cadherin protein expression and lowest levels of vimentin protein expression among three cell lines, whereas MDA-MB-231 showed the lowest levels of E-cadherin protein expression (Fig. **4A** and **B**). Moreover, by comparing the level of the main EMT markers with the level of DOCK1, we found that DOCK1 may be negatively correlated with E-cadherin and positively correlated with Vimentin (Fig. **4C**). After a knockdown of DOCK1, E-cadherin expression was significantly increased, whereas vimentin was significantly decreased compared to NC (Fig. **4D** and **E**). To further investigate the role of DOCK1 in BC cisplatin resistance, Western blotting was used to observe changes in the level of E-cadherin and Vimentin protein expression in DOCK1-siRNA-treated BC cells exposed to cisplatin. The results revealed that cisplatin treatment induced an increase in DOCK1 and Vimentin protein expression, as well as decreased E-cadherin expression, whereas DOCK1 siRNA restored these levels (Fig. **5A**-**D**). Collectively, these results indicate that DOCK1 siRNA inhibits EMT.

### 3.4 DOCK1 Knockdown Enhances the Sensitivity of BC Cells to Cisplatin *via* Twist-mediated EMT

**Fig. (6):**
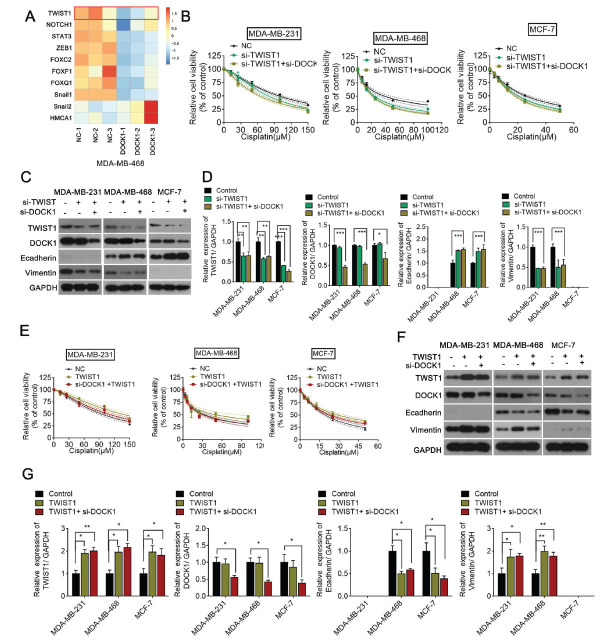
DOCK1 knockdown promotes cell sensitivity to cisplatin *via* Twist. (**A**) Heatmap of EMT-associated genes in response to DOCK1 siRNA. (**B**) A CCK-8 assay was performed to determine the viability of three breast cancer cell lines transfected with Twist siRNA or in combination with DOCK1 siRNA. (**C** and **D**) Western blotting was used to measure the level of DOCK1, Twist, E-cadherin, and vimentin protein expression in three breast cancer cell lines. **P < 0.01; ***P < 0.001. (**E**) A CCK-8 assay was performed to determine the viability of three breast cancer cell lines transfected with the Twist plasmid or in combination with DOCK1 siRNA. (**F** and **G**) Western blotting was used to measure the level of DOCK1, Twist, E-cadherin, and vimentin protein expression in three breast cancer cell lines.

Since DOCK1 was associated with EMT, we next hypothesized that DOCK1 promotes cisplatin resistance by regulating EMT. To test this hypothesis, we transfected the BC cells with DOCK1 siRNA or negative siRNA as a negative control. When analyzing the EMT-associated genes by comparison to negative control, RT-PCR revealed that Twist was significantly down-regulated in siRNA-treated cells (Fig. **6A**). To confirm that DOCK1 inhibition enhances cell sensitivity to cisplatin *via* EMT, we used Twist siRNA or a Twist plasmid to block EMT or promote EMT. Twist siRNA alone could increase the response of BC cells to cisplatin; however, when co-transfected with both Twist and DOCK1 siRNA, no significant difference in cell viability was observed between the Twist siRNA alone group and the Twist siRNA plus DOCK1 siRNA group (Fig. **6B**). A Western blot assay showed that a Twist knockdown suppressed Vimentin expression and promoted E-cadherin expression; however, the combination with DOCK1 siRNA exhibited no further effect on EMT-related proteins (Fig. **6C** and **D**). Conversely, Twist overexpression inhibited cisplatin sensitivity, whereas DOCK1 siRNA did not reverse the effect of the Twist plasmid (Fig. **6E**). Similarly, Twist upregulation facilitated EMT, while the downregulation of DOCK1 did not reverse Twist-induced EMT (Fig. **6F** and **G**). Taken together, these results demonstrate that a DOCK1 knockdown enhances the sensitivity of BC cells to cisplatin *via* Twist-mediated EMT.

## 4 DISCUSSION

In this study, we provide evidence that a DOCK1 knockdown and its inhibitor sensitize BC cells to cisplatin. Moreover, the combination of TBOPP and cisplatin increased both the *in vitro* and *in vivo* anti-cancer effects. Mechanistically, a DOCK1 knockdown can sensitize BC cells to cisplatin *via* TWIST-mediated EMT.

Cisplatin is a widely used nonspecific cell cycle phase agent for the treatment of several tumors, including lung, ovarian, and BC [25-27]. Cisplatin covalently binds with purine n-7 atoms on DNA to form DNA adducts, which distort DNA conformation, inhibit replication and transcription, and lead to an arrest of the cell cycle arrest and apoptosis by activating multiple signaling pathways [28]. Since not all patients benefit from cisplatin therapy, more effective therapeutic options are required. In BC, several therapeutic options have been reported to enhance BC cell sensitivity to cisplatin. For example, LncMT1JP has been reported to enhance cisplatin sensitivity by competitively binding to miR-24-3p in BC cells [29]. The inhibition of autophagy has been shown to enhance cisplatin-induced apoptosis in BC cells [30] markedly. A combination of hyperthermia and thermosensitive cisplatin liposomes can improve cisplatin efficacy in triple-negative BC [31]. Moreover, STAT3, but not HIF-1α, plays an important role in hypoxia-induced chemoresistance in BC cancer [32].

DOCK1 or DOCK180 was initially considered a CRK partner and was further demonstrated to activate Rac [33, 34]. DOCK1 is essential in multiple biological processes involving cell invasion and migration, cell growth, cell survival, and Akt activation [35-37]. In glioma, DOCK1 and engulfment and cell motility 1 (ELMO1) were reported to promote human glioma cell invasion [35]. The DOCK1/ELMO complex mediates Rac activation and lamellipodia formation in HeLa cells, which further promotes cell spreading and migration [38]. A recent study showed that DOCK1 is a critical regulator of HER2-mediated BC metastasis and provides a therapeutic method for limiting the spread of metastatic BCs [39]. In terms of drug resistance, previous studies have demonstrated that enoxaparin sensitizes human non-small-cell lung carcinomas to Gefitinib by inhibiting DOCK1 expression [37]. Together, these findings suggest that DOCK1 plays a carcinogenic role in tumors. In our study, we confirmed that a DOCK1 knockdown could sensitize the BC cells to cisplatin, which was similar to that of previous studies [15]. Moreover, we also confirmed that a DOCK1 inhibitor has a similar effect. TBOPP can not only sensitize the BC cells to cisplatin but also enhance the anti-tumor effect of cisplatin and inhibit tumor growth.

Recently, the role of EMT in cisplatin resistance has been increasingly recognized [40, 41]. In this study, we found that cisplatin altered EMT, as evidenced by increased E-cadherin and decreased Vimentin, suggesting that cisplatin treatment could induce EMT in BC. TWIST is one of the primary transcription factors that can regulate the EMT process. The study by Li *et al.* reported that a knockdown of TWIST increases the cytotoxicity of chemotherapeutic drugs in HepG2 cells [23]. Similarly, EGFR inhibition decreases tamoxifen resistance *via* downregulating TWIST in BC cells [42]. Here, we found that TWIST was significantly downregulated in DOCK1 siRNA-treated cells. Thus, we hypothesized that DOCK1 knockdown could sensitize BC cells to cisplatin by downregulation of Twist. Using CCK-8 and Western blotting, when Twist siRNA was used to interfere with the cells, no significant difference was observed between the Twist siRNA group and the Twist siRNA plus DOCK1 siRNA group regarding cell viability and change in EMT-associated proteins. Conversely, when the Twist plasmid was used to interfere with cells, a DOCK1 knockdown did not reverse the effect of Twist overexpression. Together, these results confirm that DOCK1 inhibits BC cell sensitivity to cisplatin through EMT.

## CONCLUSION

Our study demonstrates that TBOPP, a selective DOCK1 inhibitor, sensitizes BC cells to cisplatin *via* TWIST-mediated EMT, and the schematic mechanism is shown in Supplementary Fig. (**S1D**). Moreover, TBOPP enhances the anti-cancer effect of cisplatin. These findings demonstrate that DOCK1 may act as a potential therapeutic target in BC, and the combination of cisplatin and TBOPP may provide a promising therapeutic strategy for cisplatin-resistant BC patients.

## Data Availability

The authors confirm that the data supporting the findings of this study are available within the article and its supplementary materials.

## LIST OF ABBREVIATIONS

BC: Breast Cancer
DOCK: Dedicator of Cytokinesis
EMT: Epithelial-mesenchymal Transition
MDR: Multidrug Resistance
TBOPP: (2-(30-(trifluoromethyl)-[1,10-biphenyl]-4-yl)-2-oxoethyl)-5-pyrrolidinylsulfonyl2(1H)-pyridone
NC: Negative Control
