# Supplementary material for: TBOPP, a DOCK1 Inhibitor, Potentiates Cisplatin Efficacy in Breast Cancer by Regulating Twist-mediated EMT
Source: Curr Cancer Drug Targets. 2024 Feb 26;25(1):72–82. doi: 10.2174/0115680096281231240202073558 (PMC11826914; doi:10.2174/0115680096281231240202073558)

Supplementary Material

TBOPP, a DOCK1 Inhibitor, Potentiates Cisplatin Efficacy in Breast Cancer by Regulating Twist-mediated EMT

Xin Chen[1,#], Zhenbang Zhou[2,#], Pengting Tang[3], Feiya Du[4], Shuqian Wang[5], Jia Yao[5], Shufen Zhang[6], Jiajing Huang[6], Xuemei Lu[6], Wei Chen[6], Xiaofang Yu[2,7,*], Yu Liu[5,*] and Hao Liu[6,*]

[1]Department of Surgery, Women's Hospital, School of Medicine, Zhejiang University, Hangzhou, Zhejiang, 310006, China; [2]Cancer Institute (Key Laboratory of Cancer Prevention and Intervention, China National Ministry of Education), The Second Affiliated Hospital, Zhejiang University School of Medicine, Hangzhou, Zhejiang, 310009, China; [3]Department of Surgery, Ninghai Maternity and Child Health Hospital, Ninghai, Zhejiang, 315600, P.R. China; [4]Department of Orthopaedics, the First Affiliated Hospital, School of Medicine, Zhejiang University, Hangzhou, 310003, China; [5]Department of General Surgery, the First Affiliated Hospital, School of Medicine, Zhejiang University, Hangzhou, 310003, China; [6]Cancer Institute of Integrated traditional Chinese and Western Medicine, Key Laboratory of Cancer Prevention and Therapy Combining Traditional Chinese and Western Medicine of Zhejiang Province, Zhejiang Academy of Traditional Chinese Medicine, Tongde Hospital of Zhejiang Province, Hangzhou, 310012, Zhejiang Province, China; [7]Cancer Center, Zhejiang University, Hangzhou, Zhejiang, 310058, China

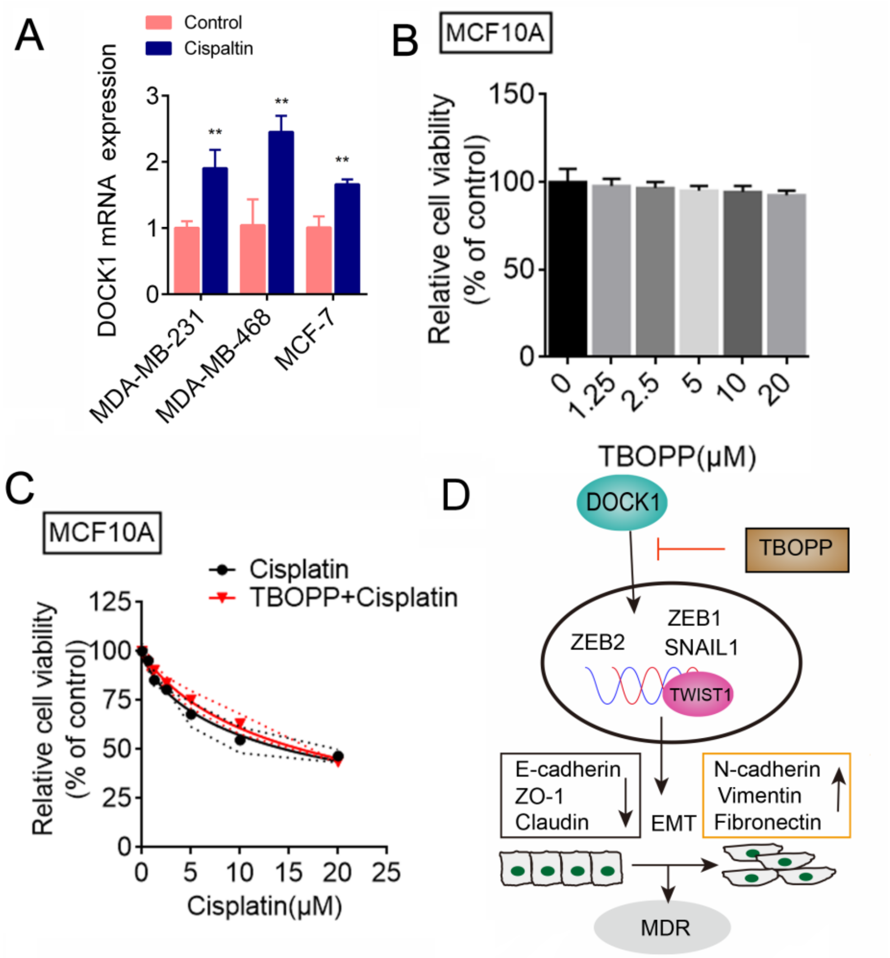

Supplement: Supplementary file 1 [file CCDT-25-1-72_SD1.pdf]
